# Food dependant exercise induced anaphylaxis a retrospective study from 2 allergy clinics in Colombo, Sri Lanka

**DOI:** 10.1186/s13223-015-0089-6

**Published:** 2015-07-25

**Authors:** Nilhan Rajiva de Silva, Wasala Mudiyanselage Dhanushka Kumari Dasanayake, Chandima Karunatilleke, Gathsauri Neelika Malavige

**Affiliations:** Department of Immunology, Medical Research Institute, Colombo, 08 Sri Lanka; Department of Microbiology, Faculty of Medical Sciences, University of Sri Jayewardenapura, Nugegoda, Sri Lanka

**Keywords:** Anaphylaxis, FDEIA, Wheat, Food allergy, Exercise

## Abstract

The aetiology of anaphylaxis ranges from food, insect venom, drugs and various chemicals. Some individuals do not develop anaphylaxis with the offending agent unless ingestion is related temporally to physical exertion, namely food dependent exercise induced anaphylaxis (FDEIA). The foods implicated are wheat, soya, peanut, milk and sea food. A retrospective study on patients with FDEIA from two Allergy clinics in Sri Lanka from 2011 to 2015 is reported. Patients were selected who fulfilled the following criteria: clinical diagnosis of anaphylaxis according to the World Allergy Organization (WAO) criteria, where the onset of symptoms was during exertion, within 4 h of ingesting a food, the ability to eat the implicated food independent of exercise, or exercise safely, if the food was not ingested in the preceding 4 h and an in vitro (ImmunoCap serum IgE to the food) or in vivo (skin prick test) test indicating evidence of sensitivity to the food. There were 19 patients (12 males: 7 females). The ages ranged from 9 to 45 (mean 22.9, median 19 years). Eight patients (42.1%) were in the 9–16 age group. Those below 16 years had a male:female ratio of 3:5, while for those above 16 years it was 9:2. Wheat was the only food implicated in FDEIA in all patients and was confirmed by skin prick testing, or by ImmunoCap specific IgE to wheat or ω − 5 gliadin. All patients had urticaria, while 5/19 (26.3%) had angioedema of the lips. Fifteen patients (78.9%) had shortness of breath or wheezing, while 8 (42.1%) had lost consciousness. Nine patients (47. 3%) had hypotension. Fourteen (73.6%) of our patients had severe reactions, with loss of consciousness or hypotension, while 5 (26.3%) had symptoms related to the gastrointestinal tract. One patient developed anaphylaxis on two occasions following inhalation of ganja, a local cannabis derivative along with the ingestion of wheat and exertion. Wheat is the main food implicated in FDEIA in Sri Lanka. A local cannabis derivative, ganja has been implicated as a cofactor for the first time.

## Background

Anaphylaxis is a potentially fatal, systemic hypersensitivity reaction [1]. The aetiology of anaphylaxis ranges from food, insect venom, drugs and various chemicals. In some cases of anaphylaxis, the individual does not develop anaphylaxis with the offending agent unless ingestion is related temporally to physical exertion [2]. The initial case report described a patient who developed anaphylaxis during exertion after ingesting shellfish [3]. This condition was termed food dependent exercise induced anaphylaxis (FDEIA) [4]. A number of food items have been implicated in FDEIA such as wheat, soya, peanut, milk and sea food [5]. Anaphylaxis following exertion, without concomitant intake of food was also described, termed exercise induced anaphylaxis (EIA) [6]. EIA constitutes 5–15% of all cases of anaphylaxis [5]. A third or half of EIA are due to FDEIA [2].

In FDEIA, anaphylaxis develops only if a specific food, or in some instances, any food, is ingested up to 4 h before exertion. In some instances, ingestion of the food may be after exertion [2]. Ingesting the food without exertion, or exertion in the absence of preceding ingestion of food does not lead to symptoms. Although the exact pathogenesis of FDEIA is not clear, changes in serum osmolality and the pH, changes in permeability of the intestinal epithelium and blood flow re distribution are thought to play a role [7]. It is believed that co factors may influence the process in two ways, by either increasing bioavailability of the food by increasing intestinal permeability or by reducing the threshold for mast cell degranulation [8]. Exercise, alcohol and certain drugs have been shown to increase the intestinal absorption of allergens, by inducing a leakage of the intestinal barrier [8], and exercise provocation has shown a dose dependent reactivity [9]. It is well documented that exercise may reduce the threshold for mast cell and basophil activation, even though the exact mechanism is still unclear [8]. Increased plasma osmolality, or activation of intestinal tissue transglutaminase (tTG) have been postulated. An increase in IL 6 (increased 50–100 times in marathon runners) upregulates tTG, which then causes aggregation of ω − 5 gliadin, a wheat component implicated in FDEIA. The aggregated product can more efficiently cross link Fcε receptors on mast cells and basophils [10].

FDEIA has been described in the West [11], and in South East Asia, including Japan [12, 13], Korea [14], Singapore [15] and Thailand [16]. Here we report for the first time, a series of cases of FDEIA due to wheat allergy in Sri Lanka.

## Case presentations

### Methods

This is a retrospective review of patients diagnosed at 2 allergy clinics in Colombo, one at the Medical Research Institute (RdeS), and the other at Asiri Surgical Hospital (GNM) between 2011 and 2015.

FDEIA was diagnosed if the patients fulfilled the criteria presented in Table 1 [1–3].

**Table 1:** Criteria for diagnosis of FDEIA [1–3]

1. Clinical diagnosis of anaphylaxis according to the World Allergy Organization (WAO) criteria [1]	
2. Onset of symptoms during exertion, within 4 h of ingesting the implicated food	
3. Ability to eat the implicated food independent of exercise, or exercise safely, if the food was not ingested in the preceding 4 h	
4. In vitro (ImmunoCap serum IgE to the implicated food) or in vivo (skin prick test) evidence of sensitivity to the food	

Case records were analysed, including clinical history and results of skin prick testing and in vitro testing for the implicated food. As this is a retrospective study, ethical clearance was obtained to collect data from the Ethics Committee of the Medical Research Institute, Colombo. However, the patients were traced and written informed consent was obtained for publication.

## Results

Nineteen patients were diagnosed with FDEIA. All had wheat dependent exercise induced anaphylaxis. No other food was identified to cause FDEIA in the two clinics during the study period, even though two patients, in addition to wheat dependent exercise induced anaphylaxis, developed anaphylaxis without ingesting wheat on 1 and 2 occasions, respectively (Table 2).

**Table 2:** Clinical Characteristics of the patients

No	Age (years)	Sex	Food	Exertion	Latent period	Clinical				Skin prick test for wheat (Positive—wheal >3 mm diameter over negative control)	ImmunoCap results For wheat kUA/L (>0.35 positive)
						Skin	Respiratory	Cardiovascular	GIT		
01	11	F	(1). Biscuit	Playing	15 min	U	S			8 mm	ND
			(2). 4 other episodes with wheat								
02	29	M	(1). Chicken sandwich	Cycling	60 min	Pr, U	S	LOC	AP	ND	IgE to wheat 12.1
			(2). 1 other episode, trigger not certain								
03	25	M	(1). Vegetable rotti*	Badminton	30 min	U		LOC		ND	IgE to wheat 2.06
			(2, 3). Pizza (x2)	Walking (x2) 1.5 and 2 km		U					
			(4). Noodles*	Dancing	30 min	U		LOC, H			
04	10	M	(1). Fishbun	Playing	30 min	U		LOC, H, no Pulse		ND	IgE to wheat 2.33
05	20	M	(1). Rotti*	Walking	60 min	U, A	S	H		ND	IgE to wheat 5.6
06	42	F	(1). Rotti*	Cleaning	15 min	U, A	W		AP	ND	IgE to wheat 2.8
			(2). Bread	Walking	30 min	U, A	W		V		
07	12	M	(1). Cream bun	Cycling, sweeping	120 min	U	W	LOC	AP, V	ND	IgE to wheat 8.3
			(2). Cake	Cycling 7 km	60 min	U, A					
			(3–10). No food	X 7 playing		U					
08	14	F	(1). Rotti*	Volleyball	15 min	U	S	LOC		3 mm	IgE to wheat 0.07
			(2). Egg bun	Walking	30 min	U					
09	14	F	(1). Sandwich	Football	45 min	U, A	S	LOC		5 mm	IgE to wheat 0.07
			(2). Pasta	Climbed 2 flights of steps on a rock	30 min (after 5 min at rest)	U					
10	11	M	(1). Chinese roll, cake	Playing	30 min	U	W		V, AP	5 mm	ND
11	42	M	(1). Rotti*	Walking	30–45 min	U	S, W			ND	IgE to wheat 5.47
12	45	M	(1). Noodles*	Walking rapidly and cleaning garden	30 min	U			AP, V	4 mm	ND
13	16	F	(1). No food	Swimming (x2)		U				5 mm	IgE to wheat 11.4
			(1). Fish bun	Badminton	20 min	U	S	Faintness, H			
14	19	M	(1). Paratha* + Ganja (cannabis for the first time)	Walked 30 min	30 min	U	S	Faintness BP unrecordable			IgE to ω − 5-gliadin 1.63
			(2). Rotti* + ganja	Football	30 min	U	S Bilateral rhonchi	Dizzines, reduced BP Anaphylactic shock			
15	18	M	(1). Kotthu	Walked 1 km	1 h (15 min after walk)	U	S			3 mm	ND
			(2). Doughnut	Exercise	4 h (1 hour after exertion)	U	S	D, H			
			(3). Paratha*	Orbitract (1 km)		U					
16	9	F	(1). Noodles*, cake, biscuits, bread, roti x9 times)	No exertion	30 min after ingestion	U					IgE wheat ω − 5-gliadin 35.7
			(2). Wafers, cookies	Played	30 min	U	W, R	D, BP 80/58 mm Hg			
17	26	M	(1). Fish bun	Cycling 3 km	30 min	U	S	Faintness			IgE wheat ω − 5-gliadin 10.6
			(2). Rice and curry. Later, wheat based pastry (Wadé*) after walk	Walked 100 yards.	10 min after eating pastry	U					
			(3). Rice, coconut sambol, eggs (breakfast)—8 am	Household chores for 1 ½ h	5 min after lunch	U		Blurring of vision, LOC			
			Rice, fish, dhal (lunch) 12.30 pm	2 h after breakfast (10–11.30 am)			S				
18	44	M	(1). Rotti*	Walked 1 km	25 min	U		Faintness, H			IgE wheat ω − 5-gliadin 3.4
19	27	F	(1). Rotti*	Walked 10 min up a hill	90 min	U, A		P, H			IgE wheat ω − 5-gliadin 5.4
			(2). Noodles*	Running 15 min	45 min	U, A	W	H			
			(3). Bun	No exertion		U					
			(4). Green gram	Gardening for 30 min	120 min	U	W	H			
			(5). Bun	Climbing hill for 10 min	15 min	U		H, LOC			
			(6). Sandwich	Exertion for 15 min	60 min	U		P, H			
			(7). Rice, carrot, potato, beans	Exertion	240 min	U		P			
			(8). Chinese roll	Exertion	15 min	U	W	P, H, LOC			
			(9). Roll	Exertion		U		LOC			

*U* urticaria, *A* angioedema, *AP* abdominal pain, *BP* blood pressure, *D* dizziness, *H* hypotension, *LOC* loss of consciousness, *P* palpitations, *Pr* pruritus, *R* rhonchi, *S* shortness of breath, *V* vomiting, *W* wheeze.

* Wheat based food: Rotti = wheat, scrapped coconut and salt containing flat bread; Paratha = wheat, coconut oil and salt containing unleavened flat bread; Kotthu = wheat, vegetables, chicken/beef containing dish; Wadé = Wheat based vegetable pastry.

Skin prick testing (SPT) was carried out in 7/19 patients, and all seven were found to be sensitized to wheat. Wheat specific IgE was determined by ImmunoCap in 15 patients: 10 with whole wheat and 5 with ω − 5-gliadin as it was only available from 2014. Eight out of 10 patients tested with whole wheat ImmunoCap (80%) gave positive results. All 5 patients tested with ω − 5-gliadin were positive. In 2/10 patients, where the whole wheat ImmunoCap gave a negative result, the SPT was positive. In one patient, both tests were positive.

Of the 19 patients, 12 were males and 7 females. The ages ranged from 9 to 45 (mean 22.9, median 19 years). Eight patients (42.1%) were in the 9–16 age group, 5 in the 20–29 age group, and only 4 were in the 41–45 age group. Those below 16 years had a male: female ratio of 3:5, while for those above 16 years it was 9:2. Seventeen patients had not developed any symptoms when ingesting wheat, prior to the development of anaphylaxis. One patient (No. 16) gave a history of developing urticaria following ingestion of wheat; however, on one occasion, the ingestion of a wheat based product followed by exertion resulted in anaphylaxis. Another patient (No. 19) could eat wheat without developing symptoms, except on one occasion, when ingestion of a bun resulted in urticaria. However, it is possible that another allergen was involved. Two patients (No. 7, 13) developed urticaria following exertion alone on 7 and 2 occasions respectively, but anaphylaxis developed when the exertion was preceded by the ingestion of wheat.

One patient (No. 19), developed anaphylaxis on 6 occasions following ingestion of a wheat based product, followed by exertion. On 2 occasions, food other than wheat also resulted in anaphylaxis, when the intake was followed by exertion. However, there have been occasions where ingestion of wheat followed by exertion did not result in anaphylaxis. On 6 of the 8 occasions where anaphylaxis had developed, the patient was having her monthly periods. Another patient developed anaphylaxis, 5 min after ingesting a meal which did not contain wheat. He had finished exertion an hour before the meal. He subsequently developed anaphylaxis following ingestion of wheat. The onset was 5 min to 4 h after ingesting a meal (mean 51.6 min, median 30 min). Fourteen patients (73.6%) reported symptoms within 30 min of eating wheat based food. The episodes occurred during exertion in 18 patients. In one patient, anaphylaxis developed 15 and 60 min after exertion. The exertions ranged from mild (domestic cleaning/sweeping) to moderate/severe (badminton, football and volleyball). The rigorousness of the exertion played a part in the clinical symptoms in three patients. One patient developed urticaria (No. 9) when resting for 5 min after mild exertion (climbing 2 flights of stairs situated on a rock) previously; subsequently she had anaphylaxis after more severe exertion (football). A second patient (No. 3) had 2 episodes of urticaria while walking (1.5 and 2 km), while he had 2 episodes of anaphylaxis after severe exertion (badminton, dancing). A third patient (No 0.13) had developed urticaria while swimming on 2 occasions, and anaphylaxis after badminton.

One patient (No. 14) had eaten wheat based food and exerted without symptoms. However, on the first occasion he had smoked ganja (local cannabis), he had ingested wheat containing food and walked for 30 min and developed anaphylaxis. On a second occasion, he had smoked ganja, ingested wheat and played football with similar results. He has smoked ganja without any symptoms subsequently provided wheat was not consumed in relation to exertion.

All patients had urticaria, while 5/19 (26.3%) had angioedema of the lips. Fifteen patients (78.9%) had shortness of breath or wheezing, while 8 (42.1%) had lost consciousness. Nine patients (47.3%) had hypotension. Fourteen (73.6%) of our patients had severe reactions, with loss of consciousness or hypotension, while 5 (26.3%) had symptoms related to the gastrointestinal tract.

There was no association with stress, humidity, cold weather, or use of non steroidal anti inflammatory drugs. However, one patient developed anaphylaxis on 6 occasions when FDEIA was during menstruation, whereas she could eat the wheat based food and exercise without symptoms at other times.

Eleven patients had only one episode, while one patient each had 8 (No. 19) and 5 episodes (No. 1) of anaphylaxis respectively. Among the group, one patient had 7 previous episodes of urticarial.

## Discussion

During a 2 ½ year period, FDEIA was diagnosed in 19 patients, who had consulted two Immunologists, serving two allergy clinics in Colombo, Sri Lanka. These clinics cater to patients from the entire island. All patients with FDEIA were allergic to wheat. Two of the patients, in addition, developed anaphylaxis following exertion after eating food other than wheat. Wheat is the commonest food responsible for FDEIA in Japan [12], Korea [14] and Thailand [16]. However, the genetic makeup is different in these countries. Shellfish has been reported to be the commonest allergen implicated in FDEIA in Singapore [17], but wheat is also being described [15]. The situation is different in the west, where tomatoes, cereal and peanuts are the commonest foods implicated in FDEIA [11]. The geographical variation may be due to the use of wheat flour in popular dishes [15], such as hoppers, string hoppers and the increasing consumption of western foods, such as sandwiches and fast foods among the urban population in Sri Lanka, replacing or supplementing rice based meals.

In a study among junior high school students in Japan, the male: female ratio was 11: 2 [13]. Eight of our patients (42.1%) were aged 9–16 years, and in contrast, the male to female ratio was 3:5. However, when patients over 16 were considered, the male: female ratio was 9: 2. The reason for the discrepancy is unclear. In the total cohort, the male: female ratio was 12:7, similar to an European study which included children as well as adults, the male: female ratio being 31:23 [11].

All patients had skin manifestations, and respiratory manifestations were seen in the majority (15/19). This is in agreement with other studies [13, 16, 18]. Eight out of 19 (42.1%) lost consciousness. Fourteen (73.6%) of our patients had severe reactions, with loss of consciousness or hypotension. Severe reactions, with hypotension, has been reported in the majority of patients (60–77.7%) in some [16, 18] but not all (25%) studies [13]. Only 5/19 patients developed gastrointestinal symptoms, similar to other studies [16].

Eighteen patients developed anaphylaxis during exercise. One patient (patient no. 15) developed anaphylaxis 15 min and 1 h, respectively, after completion of the exercise. Anaphylaxis may occur soon after exertion and it is recommended that wheat should not be ingested up to 1 h after exertion [7]. Two patients (patient no. 7, 13) developed urticaria during exertion, without the ingestion of wheat containing food; the intake of wheat resulted in anaphylaxis.

Exercise increases absorption of allergens from the gastrointestinal tract and induces mast cell degranulation. More intense exercise may provoke a more severe allergic reaction [19]. Two patients (patient no. 3, 8) developed urticaria with mild exertion (climbing 2 flights of stairs, walking 1.5 km); another (patient no. 13) developed urticaria while swimming. However, more vigorous activity (badminton, dancing, football) resulted in anaphylaxis.

All patients developed anaphylaxis during or after exertion, with wheat being ingested prior to the exercise. However, one patient (patient no. 17) developed anaphylaxis after eating wheat following exertion, on one occasion. On a previous occasion, he had developed anaphylaxis while cycling. FDEIA may occur, rarely, if food is ingested soon after exercise [2].

One co factor was noted in a patient during the episodes of FDEIA. This patient (no. 14) developed severe FDEIA if ganja (locally produced cannabis) was smoked. This happened on two occasions. The mere ingestion of wheat, even if associated with exertion did not give rise to anaphylaxis. To the best of our knowledge, this is the first report of cannabis as a co factor in FDEIA. Cannabis contains many compounds, including at least 60 cannabinoids, which are active components of cannabis. Cannabinoids act via the CB1 and CB2 receptor [20]. The CB2 receptor is found on immune and other cells, and when stimulated leads to immune cell migration and cytokine release both in the central nervous system, and peripherally [21]. Cytokine release may be a possible mechanism for cannabis acting as a cofactor in this patient. However, there are many more compounds and receptors implicated in the diverse activities of cannabis. For example, cannabidiol, a prominent psychoinactive component of cannabis has been shown to activate a rat basophil leukaemia mast cell line in a Ca^2+^ dependent manner alone or together with FcεRI stimulation, bypassing the CB 1 and CB 2 receptors [22]. Cannabis has also been implicated, albeit rarely, in IgE mediated allergic reactions [23]. Drugs that can evoke IgE mediated symptoms may function as a co factor in anaphylaxis, such as iodinated contrast media and muscle relaxants [8]. We did not do skin prick testing for cannabis in this patient due to the legal issues involved.

The other co factors implicated in FDEIA are humidity/cold, stress, menstruation and use of non steroidal anti inflammatory drugs [11]. However, Sri Lanka, a tropical country, is humid throughout the year except in the temperate central mountains. Interestingly, none of our patients were from the central hills. One patient developed anaphylaxis on 8 occasions, 6 of which occurred during menstruation. She had ingested wheat products and exercised on a few occasions without symptoms; menstruation may be considered a co factor in her situation.

The patients were diagnosed on the basis of the clinical history, and results of in vitro testing for wheat specific IgE or ω − 5-gliadin by ImmunoCap, and with skin prick testing. Eight out of 10 patients tested (80%) had ImmunoCap IgE levels to whole wheat above the cut off level (kUA/L >0.35 positive). Five patients tested positive for ω − 5 gliadin. In one study, wheat Cap detected only 41% of patients with wheat dependent exercise induced anaphylaxis [24]. Wheat protein contains salt soluble albumins and globulins, and insoluble glutens. Of the glutens, the gliadins are soluble in 70% ethyl alcohol, while the glutenins are not. Using immunoblotting, the wheat proteins ω − 5 gliadin and high molecular weight glutenins were found to be the major antigens responsible for wheat dependent EIA [25]. Using recombinant ω − 5 gliadin protein as an Immunocap, its sensitivity for wheat dependent EIA was 80%, compared to 48% for the wheat ImmunoCap [26]. In our study, 5/5 patients tested positive.

The Gold standard for the diagnosis of FDEIA is a challenge test. A standard 3 day challenge protocol has been used [16]. Due to logistic reasons as well the risk involved, we did not attempt challenge testing in our patients.

A number of hypothesis have been postulated regarding the pathophysiology of FDEIA, including increased allergen absorption from the gastrointestinal system during exercise; increase in osmolality in the villus bases activating mast cells; and blood redistribution to skin and muscle exposing sensitive mast cells in these areas to food allergens [2].

All patients were advised to avoid ingesting wheat based food 4 h before and one hour after exercise [2]. Some patients were prescribed the autoinjectable adrenaline pen (“Epipen”). Many patients were unable to afford this device as it is not supplied free of charge by the Ministry of Health. Attempts are being made to supply this vital drug, on a named patient basis, to patients at risk of anaphylaxis.

## Conclusion

Wheat is the main food item implicated in FDEIA in Sri Lanka. A local cannabis product, ganja, was implicated as a co factor in one patient, the first report in the literature. Lack of awareness of FDEIA may lead to an inaccurate diagnosis by the physician and the patient may be placed on unnecessary dietary restrictions and exercise. Therefore, we feel that all the primary care physicians must be educated on this important disease entity.

## Consent

Case records were analysed, including clinical history and results of skin prick testing and in vitro testing for the implicated food. As this is a retrospective study, ethical clearance was obtained to collect data from the Ethics Committee of the Medical Research Institute, Colombo. However, the patients were traced and written informed consent was obtained for publication.
